# Inguinoscrotal Hernia: An Unusual Presentation of Retroperitoneal Liposarcoma

**DOI:** 10.7759/cureus.57231

**Published:** 2024-03-29

**Authors:** Sanjay M Khaladkar, Saksham Jain, Tejvir Singh, Satvik Dhirawani

**Affiliations:** 1 Radiodiagnosis, Dr. D. Y. Patil Medical College, Hospital and Research Centre, Pune, IND

**Keywords:** dedifferentiated, inguinoscrotal, inguinal hernia, liposarcoma, retroperitoneal

## Abstract

Liposarcoma presenting within an inguinal hernia is rare. It may manifest as either primary spermatic cord liposarcoma or as an extension of retroperitoneal liposarcoma into the inguinoscrotal region. Tumors originating in the retroperitoneum can extend toward the inguinal region through the gonadal vein pathway. Utilizing imaging modalities is crucial for differentiating between a retroperitoneal component and a fat-containing inguinal mass. Identification of non-lipomatous components within a fat-containing tumor provides diagnostic clues on radiological imaging.

## Introduction

Liposarcoma in an inguinal hernia can be either a primary spermatic cord liposarcoma or an extension of retroperitoneal liposarcoma into the inguinoscrotal region. Differentiation between the two is essential preoperatively for surgical management to ensure adequate surgical resection with clear margins. Imaging modalities play an important role in ruling out the retroperitoneal component of a fat-containing inguinal mass [1]. Liposarcomas are malignant soft tissue neoplasms showing adipocytic differentiation. They affect people of all ages, from middle-aged to old, and affect both males and females equally [2]. The anatomic site, grade, and resectability determine the prognosis. Low-grade well-differentiated liposarcoma has a five-year survival rate of 85%, while high-grade liposarcoma and dedifferentiated liposarcoma have a five-year survival rate of 18%. It commonly occurs in the extremities followed by the retroperitoneum. Mortality is higher in patients undergoing hernia repair rather than oncologic resection during the initial surgery. The completeness of excision of the tumor has a good prognosis, as liposarcomas usually invade locally rather than metastasize [1].

## Case presentation

A 56-year-old male presented with swelling in the right inguinoscrotal region for the last six months. It was not associated with pain, fever, vomiting, or altered bowel habits. There were no urinary complaints and no history of weight loss, hypertension, diabetes, or tuberculosis. On examination, a hard, immobile lump, about 6 × 4 cm in size, was palpable in the right inguinal region, extending inferiorly into the right scrotum. Both testicles were palpable and appeared normal. The lump was irreducible, with no cough impulse noted. It was not possible to get above the swelling. Laboratory reports including hemogram, urine examination, renal function test, and liver function tests were normal.

The ultrasound (USG) of the inguinoscrotal region, conducted with a linear high-resolution 9-12 MHz probe, revealed a solid mass with heterogeneous echotexture (predominantly echogenic) in the right inguinal region, extending inferiorly into the right scrotal sac. In the right hemiscrotum, it compressed and displaced the right testis inferiorly. Both testicles and epididymis appeared normal. The right spermatic cord was not distinctly visible. In the right inguinal region, it extended laterally to the inferior epigastric vessels. Its superior extent was difficult to assess, with suspicion of extension into the lower abdomen. The possibility of a right inguinoscrotal hernia containing fat was raised.

**Figure 1:**
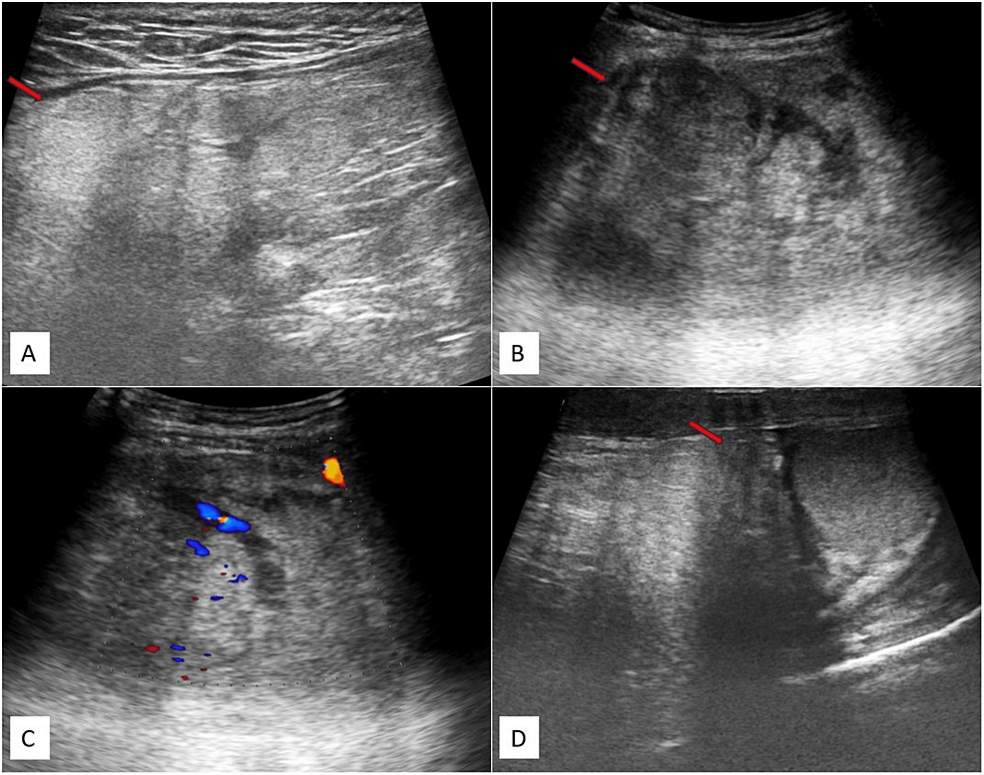
USG images (A-D) of the inguinoscrotal region depict a solid mass with heterogeneous echotexture in the right inguinal region. The mass extends into the right scrotal sac, reaching up to the upper pole of the right testes, and shows internal vascularity. The USG image reveals a solid mass with heterogeneous echotexture in the right inguinal region, where the superior margins could not be well assessed (red arrow) (A), extending into the right scrotal sac (red arrow) (B), displaying mild internal vascularity (C), and reaching up to the upper pole of the right testis (red arrow) (D). USG: ultrasonography

Figure 1 depicts ultrasonography (USG) images (Figure 1A-1D) of the inguinoscrotal region, illustrating a mass in the right inguinal area that extends into the right scrotal sac and reaches up to the upper pole of the right testes, displaying internal vascularity. Its superior margins were not well defined.

Because ultrasound (USG) had limitations in assessing the margins of the mass, plain and contrast-enhanced computed tomography (CT) of the abdomen were recommended. The scans revealed a large, well-defined solid mass in the retroperitoneum, beginning approximately 1.4 cm inferior to the level of the aortic bifurcation. It extended inferiorly along the anterior aspect of the right psoas muscle, beneath the anterior abdominal wall, and involved the right common iliac vessels. Mild extrinsic compression was noted on the medial surface of the cecum. The pelvic portion of the right ureter was compressed and displaced medially, while the rectosigmoid was compressed and displaced toward the left. The right common iliac artery was compressed from its anterior aspect, and the right external and internal iliac vessels were compressed and displaced posteriorly by the mass. The anterior abdominal wall muscles were compressed and displaced anteriorly, and the right psoas muscle was flattened. The mass exhibited predominantly fat density with a CT value of -50 to -140 Hounsfield units (HU). Multiple soft tissue density septations, measuring more than 3 mm in thickness, were noted and showed post-contrast enhancement. No calcifications were observed. Its central portion showed a soft tissue density component measuring approximately 5.3 × 8.1 × 12.2 cm (anteroposterior × transverse × craniocaudal), exhibiting heterogeneous post-contrast enhancement. The mass extended inferiorly into the pelvis along the right lateral pelvic wall, causing a mass effect on the rectosigmoid and the dome of the urinary bladder. In the inguinal region, it was anteromedial to the right pectineus muscle and lateral to the inferior epigastric vessels, extending inferiorly into the right hemiscrotum up to the upper pole of the right testis, which was compressed and displaced inferiorly. The mass had the widest dimensions of approximately 21 × 18 × 13 cm (craniocaudal × transverse × anteroposterior). In our case, displacement of the right ureter and right external and internal iliac vessels, and compression on the rectosigmoid and cecum provided clues to the retroperitoneal origin of the tumor. A diagnosis of a retroperitoneal fat-containing tumor (liposarcoma) was made, with extension into the right hemiscrotum due to a right indirect inguinal hernia. Other organs, including the liver, spleen, gallbladder, pancreas, both kidneys, and adrenal glands, appeared normal. No hepatic or adrenal metastasis, retroperitoneal lymphadenopathy, or ascites were noted.

**Figure 2:**
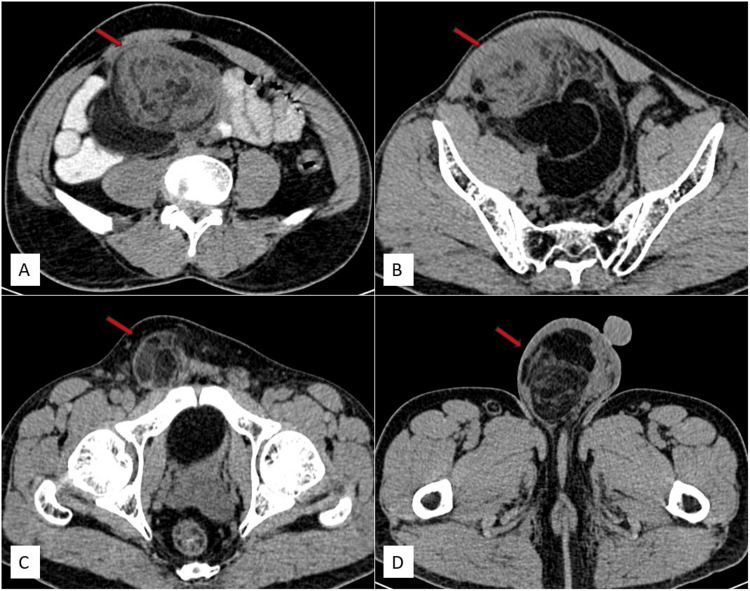
Axial non-contrast CT images depict a solid mass of heterogeneous density, showing both lipomatous and non-lipomatous components, in the retroperitoneum. It extends inferiorly into the pelvis, right inguinal region, and right scrotal sac up to the upper pole of the right testis. Axial non-contrast CT images reveal a mass of heterogeneous density in the retroperitoneum (red arrow) (A), extending inferiorly into the pelvis (red arrow) (B) and right inguinal region (red arrow) (C) and reaching the right scrotal sac up to the upper pole of the right testis (red arrow) (D). CT: computed tomography

Figure 2 displays axial non-contrast CT images (Figure 2A-2D) illustrating a mass in the retroperitoneum, extending inferiorly into the pelvis, right inguinal region, and right scrotal sac up to the upper pole of the right testes.

**Figure 3:**
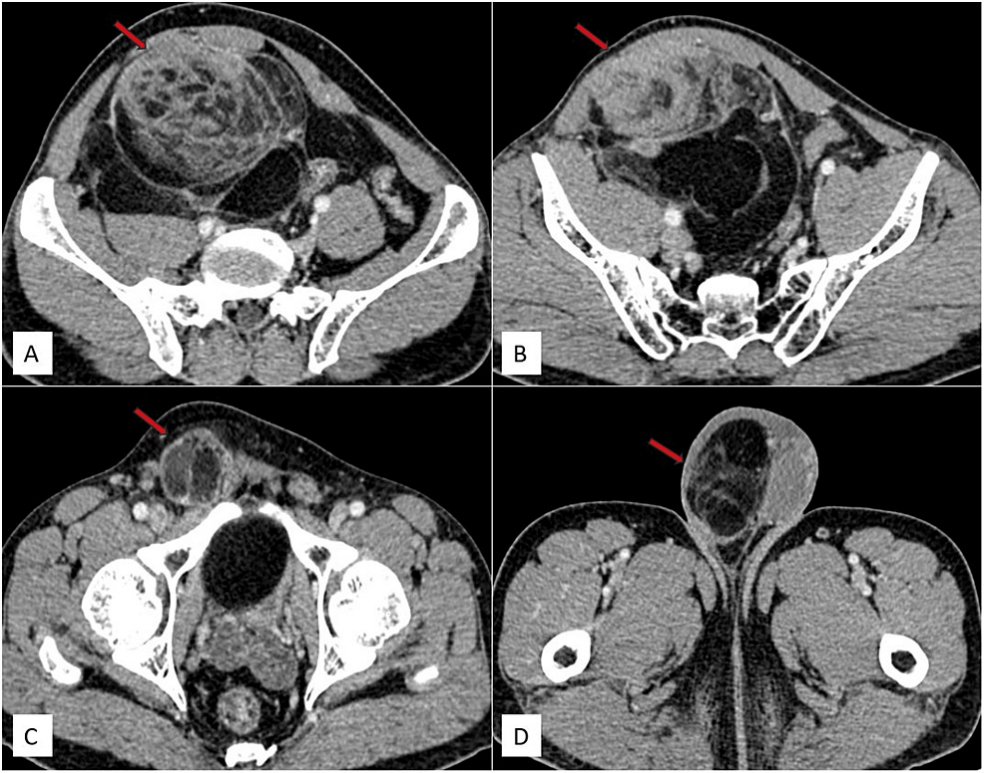
Axial contrast CT images depict a solid mass of heterogeneous density, showing both lipomatous and non-lipomatous components, in the retroperitoneum. It extends inferiorly into the pelvis, right inguinal region, and right scrotal sac up to the upper pole of the right testis. The solid component exhibits heterogeneous enhancement. Axial contrast CT images reveal a mass of heterogeneous density in the retroperitoneum (red arrow) (A), extending inferiorly into the pelvis (red arrow) (B) and right inguinal region (red arrow) (C) and reaching the right scrotal sac up to the upper pole of the right testis (red arrow) (D). CT: computed tomography

Figure 3 displays axial contrast CT images (Figure 3A-3D) illustrating a mass in the retroperitoneum, extending inferiorly into the pelvis, right inguinal region, and right scrotal sac up to the upper pole of the right testes.

**Figure 4:**
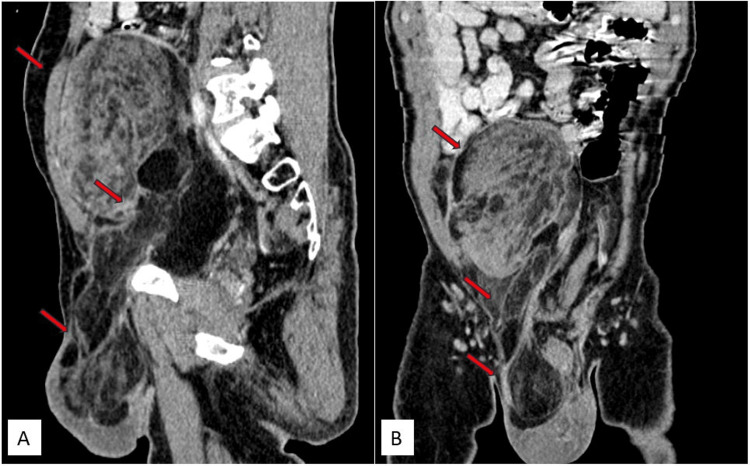
Contrast CT images (sagittal and coronal) depict a solid mass of heterogeneous density, showing both lipomatous and non-lipomatous components, in the retroperitoneum. It extends inferiorly into the pelvis, right inguinal region, and right scrotal sac up to the upper pole of the right testis. The solid component exhibits heterogeneous enhancement. Contrast CT images reveal a mass of heterogeneous density in the retroperitoneum extending inferiorly into the pelvis, right inguinal region, and right scrotal sac, reaching up to the upper pole of the right testis in sagittal (red arrows) (A) and coronal (red arrows) (B) sections. CT: computed tomography

Figure 4 displays sagittal and coronal contrast CT images (Figure 4A, 4B) illustrating a mass in the retroperitoneum, extending inferiorly into the pelvis, right inguinal region, and right scrotal sac up to the upper pole of the right testes.

**Figure 5:**
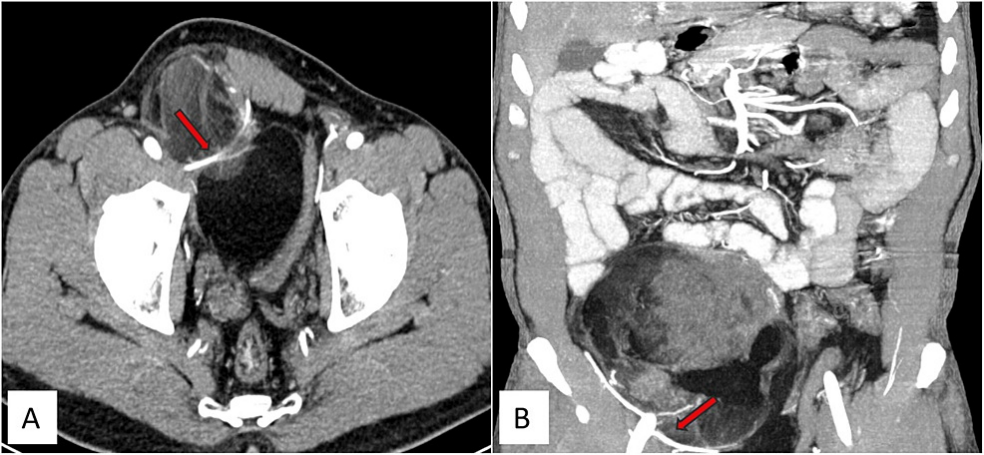
Contrast CT images in the arterial phase depicting the relation of the right inguinal mass and the inferior epigastric artery. The contrast CT scan (arterial phase) illustrates the right inguinal mass positioned laterally to the inferior epigastric artery, as indicated by the red arrow in both axial (A) and coronal (B) sections. CT: computed tomography

Figure 5 displays axial and coronal contrast CT images in the arterial phase, illustrating the relation of the right inguinal mass to the inferior epigastric artery.

Histopathological examination of the tru-cut biopsy of the swelling suggested a low-grade liposarcoma. An exploratory laparotomy was performed, involving the excision of retroperitoneal liposarcoma along with the appendix and right testis. The tumor was located in the retroperitoneum, with inferior extension via the inguinal canal into the right scrotal sac, with dimensions of approximately 21 × 18 × 13 cm (craniocaudal × transverse × anteroposterior). Intraoperatively, it presented as a dumbbell-shaped mass, with one end in the abdomen and the other in the scrotum. The larger abdominal swelling was well-circumscribed and bosselated and had a gray-yellow appearance, measuring approximately 10 × 10 × 13 cm. The scrotal swelling was smaller, bosselated, and well-circumscribed, measuring approximately 9 × 5 × 2.5 cm. The histologic type was well-differentiated liposarcoma, grade I. No necrosis was identified, and the mitotic rate was 4-5 mitoses per 10 high-power fields (HPFs). No lymphovascular invasion was detected. The pathological grade was grade I (score: 1 + 1 + 0 = 2). The appendix and right testis were free of the tumor.

## Discussion

Liposarcoma is one of the most common soft tissue sarcomas (STSs) in adults [3]. Retroperitoneal liposarcoma is often asymptomatic, and symptoms occur due to mass effects on adjoining structures such as pain, obstruction, and gastrointestinal bleeding. The etiology of retroperitoneal liposarcoma is unclear, and it does not arise from a benign lipomatous tumor. Malignant adipocytic tumors were classified by the World Health Organization (WHO) in 2020 as follows: well-differentiated liposarcoma (lipoma-like, sclerosing, and inflammatory), dedifferentiated liposarcoma, myxoid liposarcoma, pleomorphic liposarcoma, and myxoid pleomorphic liposarcoma [4].

Well-differentiated liposarcomas constitute 50% of all liposarcomas. The lower extremities (50%) are the most common location, followed by the retroperitoneum (20%) [2,5,6]. Histologically, they resemble normal adipose tissue and are composed of mature adipocytes. Adipocytes may exhibit nuclear atypia and vary in size, and lipoblasts may be present. Subcategories include lipoma-like, inflammatory, sclerosing, and spindle cell types, depending on additional features. Being a low-grade tumor compared to other types, it has the least metastatic potential but a high local recurrence rate [2].

The differential diagnosis of fat-containing lesions in inguinal hernias includes lipoma, lipomatosis, liposarcoma of the spermatic cord, omental hernia, liposarcoma, and retroperitoneal fat. Their differentiation on ultrasound is often difficult. Hence, further imaging by CT/magnetic resonance imaging (MRI) can provide clues to diagnosis [7]. The presence of a lipomatous mass with a non-lipomatous component suggests a diagnosis of well-differentiated liposarcoma on CT and MRI [5,6]. Non-lipomatous components include the presence of septa (usually more than 2 mm thick) and nodular or globular soft tissue components (usually less than 2 cm in size), which provide clues to well-differentiated liposarcoma. Contrast studies show enhancement of septae and soft tissue components, while the lipomatous component shows a lack of enhancement. Dedifferentiated liposarcoma can arise within well-differentiated liposarcomas. A large size of non-lipomatous component (>2 cm in size) provides a clue for its diagnosis. MRI, due to its soft tissue discrimination capability, can evaluate non-lipomatous components, which appear hypointense on T1-weighted imaging (T1WI) and hyperintense on T2-weighted imaging (T2WI) [5].

Retroperitoneal well-differentiated liposarcomas have a recurrence rate of more than 90%, whereas dedifferentiated liposarcomas have a recurrence rate of almost 100%. This is attributed to the difficulty in achieving negative surgical margins. Since the right gonadal vein drains directly into the inferior vena cava (IVC), the presence of right-sided varicocele is rare. Imaging modalities should be employed to rule out retroperitoneal pathology compressing the right gonadal vein in cases of right-sided varicocele [8].

Liposarcoma presenting within an inguinal hernia is rare and may signify a deeper underlying issue. The primary tumor might not be readily identifiable through clinical examination [9]. The retroperitoneal origin of the tumor is often indicated by the displacement of adjacent retroperitoneal organs or major vessels as seen on CT or MRI scans. The retroperitoneum seamlessly connects with the pelvic prevesicular space [10]. This prevesicular space serves as the pathway for the testicular vessels to enter the spermatic cord, passing through the internal inguinal ring. Prevesicular fat accompanies the testicular veins and is enveloped by the internal spermatic fascia [11].

Incarcerated omental inguinal hernia mimics well-differentiated/dedifferentiated liposarcoma extending into the inguinal canal both clinically and radiologically. Both present as a palpable groin mass that is irreducible and shows a non-lipomatous component on imaging findings. Incarcerated omental inguinal hernias show omental arteries with no relation to testicular vessels [11].

As well-differentiated liposarcomas are poorly demarcated from adjoining homogeneous low-density normal fat, they pose a diagnostic challenge. High-grade liposarcomas are solid and heterogeneous with soft tissue components, making them easier to detect. The presence of internal septations, soft tissue components, and fat stranding within a fat-containing tumor raises the possibility of liposarcoma. Liposarcomas can exhibit discontinuous lobulated growth with separate nodules, leading to a high recurrence rate despite tumor-free excision margins in prior surgical sites, with rates of recurrence reaching up to 75%. Surgical treatment involves radical high orchidectomy (with margins as close to the inguinal canal as possible) and en bloc excision of the tumor (complete resection with wide margins). Retroperitoneal lymph node dissection, radiotherapy, or chemotherapy are not typically required. Close interval follow-up with imaging is necessary to rule out recurrence [1].

Retroperitoneal liposarcoma, a rare form of cancer, typically necessitates surgery as the primary treatment. Diagnosing well-differentiated retroperitoneal liposarcoma and monitoring patients post-surgery pose considerable challenges. Recurrence following surgery is common, highlighting the importance of more frequent CT or MRI follow-ups to detect tumors at an earlier stage. The latest guidelines emphasize the necessity of a multidisciplinary approach involving experienced soft tissue sarcoma (STS) management physicians for treating primary LPS, underscoring the importance of referral to such specialists whenever feasible [12].

Although chemotherapy protocols and radiation therapy are still considered effective supplementary treatments, surgical intervention involving complete tumor removal, indicated by negative surgical margins, continues to be the preferred method. Research suggests a notable disparity in mid-term survival rates between patients who undergo adequate tumor excision and those with positive surgical margins, thereby reinforcing the superiority of surgical treatment as the optimal approach [13].

## Conclusions

Retroperitoneal lipomatous tumors may present as a small palpable inguinal mass. This occurrence resembles only a fraction of the larger issue, much like the visible tip of an iceberg, with the primary tumor frequently being overlooked and undetected through clinical means. Radiological investigations play a crucial role in diagnosing the origin of the mass and its extension into the inguinal hernia. Internal septations, soft tissue components, and fat stranding within the fat-containing tumor raise the possibility of a sarcomatous component in the lipomatous tumor, which can be detected on CT scans. CT scans are essential preoperatively for surgical management to ensure adequate surgical resection with clear margins.
